# Disinfection and Disinfectants

**Published:** 1885-04

**Authors:** George M. Sternberg

**Affiliations:** Major and Surgeon U. S. A. Chairman Committee on Disinfectants.


AMERICAN JOURNAL

OF

### DENTAL SCIENCE.

VOL. XVIII. THIRD SERIES—APRIL, 1885. No. 12.

ARTICLE I.

### DISINFECTION AND DISINFECTANTS.

**(Preliminary Report made by the Committee on Disinfectants of the American Public Health Association.**

The object of *disinfection* is to prevent the extension of infectious diseases by destroying the specific infectious material which gives rise to them. This is accomplished by the use of *disinfectants*.

There can be no partial disinfection of such material; either its infecting power is destroyed or it is not. In the latter case there is a failure to disinfect. Nor can there be any disinfection in the absence of infectious material.

It has been proved for several kinds of infectious material that its specific infecting power is due to the presence of living micro-organisms, known in a general way as "disease germs;" and practical sanitation is now based upon the belief that the infecting agents in all kinds of infectious material are of this nature. Disinfection, therefore, consists essentially in the destruction of disease germs.

Popularly, the term disinfection is used in a much broader sense. Any chemical agent which destroys or masks bad odors, or which arrests putrefactive decomposition is spoken of as a disinfectant. And in the absence of any infectious disease it is common to speak of disinfecting a foul cess-pool, or bad-smelling stable, or privy vault.

This popular use of the term has led to much misapprehension, and the agents which have been found to destroy bad odors—*deodorisers*—or to arrest putrefactive decomposition—*antiseptics*—have been confidently recommended and extensively used for the destruction of disease germs in the excreta of patients with cholera, typhoid fever, etc.

The injurious consequences which are likely to result from such misapprehension and misuse of the word disinfectant will be appreciated when it is known that:

*Recent researches have demonstrated that many of the agents which have been found useful as deodorisers, or as antiseptics, are entirely without value for the destruction of disease germs.*

This is true, for example, as regards the sulphate of iron or copperas, a salt which has been extensively used with the idea that it is a valuable disinfectant. As a matter of fact, sulphate of iron in saturated solution does not destroy the vitality of disease germs or the infecting power of material containing them. This salt is, nevertheless, a very valuable antiseptic, and its low price makes it one of the most available agents for the arrest of putrefactive decomposition in privy vaults, etc.

*Antiseptic agents also exercise a restraining influence upon the development, of disease germs, and their use during epidemics is to be recommended, when masses of organic material in the vicinity of human habitations cannot be completely destroyed, or removed, or disinfected.*

While an antiseptic agent is not necessarily a disinfectant, all disinfectants are antiseptics; for putrefactive decomposition is due to the development of "germs" of the same class as that to which disease germs belong, and the agents which destroy the latter also destroy the bacteria of putrefaction, when brought in contact with them in sufficient quantity, or restrain their development when present in smaller amounts.

*A large number of the proprietary "disinfectants," so called, which are in the market, are simply deodorisers or antiseptics, of greater or less value, and are entirely untrustworthy for disinfecting purposes.*

Antiseptics are to be used at all times when it is impracticable to remove filth from the vicinity of human habitations, but they are a poor substitute for cleanliness.

During the prevalence of epidemic diseases, such as yellow fever, typhoid fever and cholera, it is better to use, in privy-vaults, cess-pools, etc., those antiseptics which are also disinfectants—i. e., germicides; and when the contents of such receptacles are known to be infected this becomes imperative.

Still more important is the destruction at our sea-port quarantine stations of infectious material which has its origin outside of the boundaries of the United States, and the destruction, within our boundaries, of infectious material given off from the persons of those attacked with any infectious disease, whether imported or of indigenous origin.

*In the sick-room we have disease germs at an advantage, for we know were to find them as well as how to kill them.*

Having this knowledge, not to apply it would be criminal negligence, for our efforts to restrict the extension of infectious diseases must depend largely upon the proper use of disinfectants in the sick room.

### GENERAL DIRECTIONS.

*Disinfection of Excreta, etc.*—The infectious character of the dejections of patients suffering from cholera and from typhoid fever is well established; and this is true of mild cases and of the earliest stages of the diseases as well as of severe and fatal cases. It is probable that epidemic dysentery, tuberculosis, and perhaps diphtheria, yellow fever, scarlet fever and typhus fever may also be transmitted by means of the alvine discharges of the sick. It is therfore of the first importance that these should be disinfected. In cholera, diphtheria, yellow fever and scarlet fever, all vomited material should also be looked upon as infectious. And in tuberculosis, diphtheria, scarlet fever and infectious pneumonia, the sputa of the sick should be disinfected or destroyed by fire. It seems advisable also to treat the urine of patients sick with an infectious disease with one of the disinfecting solutions below recommended.

*Chloride of lime*, or bleaching powder, is, perhaps, entitled to the first place for disinfecting excreta, on account of the rapidity of its action. The following standard solution is recommended:

### STANDARD SOLUTION NO. 1.

*Dissolve Chloride of Lime of the best quality in soft water, in the proportion of four ounces to the gallon.*

Use one pint of this solution for the disinfection of each discharge in cholera, typhoid fever, etc. Mix well and leave in vessel for at least ten minutes before throwing into privy-vault or water-closet. The same directions apply for the disinfection of vomited matters. Infected sputum should be discharged directly into a cup half full of the solution.

### STANDARD SOLUTION NO. 2.

*Dissolve Corrosive Sublimate and Permanganate of Potash in soft water, in the proportion of two drachms of each salt to the gallon.*

This is to be used for the same purposes and in the same way as *Standard Solution No.* 1. It is equally effective, but it is necessary to leave it for a longer time in contact with the material to be disinfected—at least an hour.

The only advantage which this solution has over the chloride of lime solution consists in the fact that it is odorless, while the odor of chlorine in the sick room is considered by some persons objectionable. The cost is about the same.* It must be remembered that this solution is highly poisonous. It is proper, also, to call attention to the fact that *it will injure lead pipes if passed through them in considerable quantities.*

### STANDARD SOLUTION. No. 3

*To one part of Labarraque's Solution, (liquor sodæ chlorinatæ.) add five parts of soft water.*

This solution is more expensive† than the solution of chloride of lime, and has no special advantages for the purposes mentioned. It may, however, be used in the same manner as recommended for *Standard Solution No.* 1.

The following powder is also recommended for the disinfection of excreta in the sick room and of privy-vaults, etc.:

### DISINFECTING AND ANTISEPTIC POWDER.

*One pound of chloride of lime; one ounce of corrosive sublimate; nine pounds of plaster of Paris. Pulverise the corrosive sublimate and mix thoroughly with the plaster of Paris. Then add the chloride of lime and mix well. Pack in paste-board boxes or in wooden casks. Keep dry.*

As an antiseptic and deodoriser this powder is to be sprinkled upon the surface of excreta, etc.

To disinfect excreta in the sick room, cover the entire surface with a thin layer of the powder—one-fourth inch in thickness—and if the material is not liquid pour on sufficient water to cover it.

*Disinfection of the Person.*—The surface of the body of a sick person, or of his attendants, when soiled with infectious discharges, should be at once cleansed with a suitable disinfecting agent. For this purpose *Standard*

*Solution No.* 3. may be used.

In diseases like small-pox and scarlet fever, in which the infectious agent is given off from the entire surface of the body, occasional ablutions with Labarraque's Solution diluted with twenty parts of water, will be more suitable than the stronger solution above recommended.

In all infectious diseases the surface of the body of the dead should be thoroughly washed with one of the standard solutions above recommended, and then enveloped in a sheet saturated with the same.

*Disinfection of Clothing.*—Boiling for half an hour will destroy the vitality of all known disease germs, and there is no better way of disinfecting clothing or bedding which can be washed than to put it through the ordinary operations of the laundry. No delay should occur, however, between the time of removing soiled clothing from the person or bed of the sick and its immersion in boiling water, or in one of the following solutions; and no article should be permitted to leave the infected room until so treated.

### STANDARD SOLUTION NO. 4.

*Dissolve corrosive sublimate in water in the proportion of four ounces to the gallon, and add one drachm of permanganate of potash to each gallon to give color to the solution.*

One fluid ounce of this standard solution to the gallon of water will make a suitable solution for the disinfection of clothing. The articles to be disinfected must be thoroughly soaked with the disinfecting solution and left in it for at least two hours, after which they may be wrung out and sent to the wash.

N. B. *Solutions of corrosive sublimate shovld not be placed in metal receptacles*, for the salt is decomposed and the mercury precipitated by contact with copper, lead or tin. A wooden tub or earthen crock is a suitable receptacle for such solutions.

Clothing may also be disinfected by immersion for two hours in a solution made by diluting *Standard Solution No.* 1 with nine parts of water—one gallon in ten. This solution is preferable for general use, especially during the prevalence of epidemics, on account of the possibility of accidents from the poisonous nature of *Standard Solution No.* 4. When diluted as directed this solution may, however, be used without danger from poisoning through the medium of clothing immersed in it, or by absorption through the hands in washing. A poisonous dose could scarcely be swallowed by mistake, owing to the metallic taste of the solution, and the considerable quantity which would be required to produce a fatal effect—at least half a pint.

Clothing and bedding which cannot be washed may be disinfected by exposure to dry heat in a properly constructed disinfecting chamber for three or four hours. A temperature of 230° Fah. should be maintained during this time, and the clothing must be freely exposed—*i. e.*, not folded or arranged in piles or bundles, for the penetrating power of dry heat is very slight.

The limitations with reference to the use of dry heat as a disinfectant are stated in a "Preliminary Report of the Committee on Disinfectants," published in *The Medical News*, Philadelphia, March 14, 1885.

The temperature above mentioned will not destroy the *spores* of bacilli—e. g. of the anthrax bacillus, but is effective for the destruction of all disease germs which do not form spores; and there is good reason to believe that this list includes small-pox, cholera, yellow fever, diphtheria, erysipelas, puerperal fever, and scarlet fever(?) Moist heat is far more effective, and it is demonstrated that ten minutes exposure to steam, at a temperature of 230° Fah., will destroy all known disease germs including the most refractory spores.

In the absence of a suitable disinfecting chamber, it will be necessary to burn infected clothing and bedding, the value of which would be destroyed by immersion in boiling water, or in one of the disinfecting solutions recommended.

*Disinfection of the sick room.*—In the sick room no disinfectant can take the place of free ventilation and cleanliness. It is an axiom in sanitary science that *it is impracticable to disinfect an occupied apartment*; for the reason that disease germs are not destroyed by the presence in the atmosphere of any known disinfectant in respirable quantity. Bad odors may be neutralised, but this does not constitute disinfection in the sense in which the term is here used. These bad odors are, for the most part, an indication of want of cleanliness, or of proper ventilation; and it is better to turn contaminated air out of the window, or up the chimney, than to attempt to purify it by the use of volatile chemical agents, such as carbolic acid, chlorine, etc., which are all more or less offensive to the sick, and are useless so far as disinfection—properly so-called—is concerned.

*When an apartment which has been occupied by a person sick with an infectious disease is vacated, it should be disinfected.* But it is hardly worth while to attempt to disinfect the atmosphere of such an apartment, for this will escape through an open window and be replaced by fresh air from without, while preparations are being made to disinfect it. Moreover, experience shows that the infecting power of such an atmosphere is quickly lost by dilution, or by the destruction of floating disease germs through contact with oxygen, and that even small-pox and scarlet fever are not transmitted to any great distanee through the atmosphere; while cholera, typhoid fever, and yellow fever, are rarely, if ever, contracted by contact with the sick, or by respiring the atmosphere of the apartments occupied by them.

The object of disinfection in the sick room is, mainly, the destruction of infectious material attached to surfaces, or deposited as dust upon window-ledges, in crevices, etc. If the room has been properly cleansed and ventilated while still occupied by the sick person, and especially if it was stripped of carpets and unnecessary furniture at the outset of his attack, the difficulties of disinfection will be greatly reduced.

All surfaces should be thoroughly washed with a solution of corrosive sublimate of the strength of one part in 1000 parts of water, which may be conveniently made by adding four ounces of *Standard Solution No.* 4 to the gallon, or one pint to four gallons of water. The walls and ceiling, if plastered, should be brushed over with this solution, after which they may be whitewashed with a lime wash. Especial care must be taken to wash away all dust from window-ledges and other places where it may have settled, and to thoroughly cleanse crevices and out-of-the-way places. After this application of the disinfecting solution, and an interval of twenty-four hours or longer for free ventilation, the floors and wood-work should be well scrubbed with soap and hot water, and this should be followed by a second more prolonged exposure to fresh air, admitted through open doors and windows.

Many sanitary authorities consider it necessary to insist upon fumigation with sulphurous acid gas-produced by combustion of sulphur—for the disinfection of the sick room. As an additional precaution this is to be recommended, especially for rooms which have been occupied by patients with small-pox, scarlet fever, diphtheria, typhus fever and yellow fever. It should precede the washing of surfaces and free ventilation above recommended. But fumigation with sulphurous acid gas alone, as commonly practiced, cannot be relied upon for the disinfection of the sick room and its contents, including bedding, furniture, infected clothing, etc., as is popularly believed. And a misplaced confidence in this mode of disinfection is likely to lead to a neglect of the more important measures which have been recommended. In the absence of moisture the disinfecting power of sulphurous acid gas is very limited, and under no circumstances can it be relied upon for the destruction of spores But exposure to this agent in sufficient quanity, and for a considerable time, especially in the presence of moisture, is destructive of disease germs, in the absence of spores. It is essential, however, that the germs to be destroyed shall be very freely exposed to the disinfecting agent, which has but slight, penetrating power.

*To secure any results of value it will be necessary to close the apartment to be disinfected as completely as possible by stopping all apertures through which the gas might escape, and to burn not less than three pounds of sulphur for each thousand cubic feet of air-space in the room.* To secure complete combustion of the sulphur it should be placed, in powder or in small fragments, in a shallow iron pan, which should be set upon a couple of bricks in a tub partly filled with water, to guard against fire. The sulphur should be thoroughly moistened with alcohol before igniting it.

*Disinfection of privy-vaults, cess-pools, etc.* When the excreta—not previously disinfected—of patients with cholera or typhoid fever, have been thrown into a privy-vault this is infected, and disinfection should be resorted to as soon as the fact is discovered, or whenever there is reasonable suspicion that such is the case. It will be advisable to take the same precautions with reference to privy-vaults into which the excreta of yellow fever patients have been thrown, although we do not definitely know that this is infectious material. Disinfection may be accomplished either with corrosive sublimate or with chloride of lime. The amount used must be proportioned to the amount of material to be disinfected.

*Use one pound of corrosive sublimate for every five hundred pounds*—estimated—*of fecal matter contained in the vault, or one pound of chloride of lime to every thirty pounds.*

*Standard Solution No.* 4, diluted with three parts of water may be used. It should be applied—the diluted solution—in the proportion of one gallon to every four gallons—estimated—of the contents of the vault.

If chloride of lime is to be used, one gallon of *Standard Solution No.* 1 will be required for every gallon—estimated

—of the material to be disinfected.

All exposed portions of the vault, and the wood-work above it, should be thoroughly washed down with the disinfecting solution.

To keep a privy-vault disinfected during the progress of an epidemic, sprinkle chloride of lime freely over the surface of its contents daily. Or, if the odor of chlorine is objectionable, apply daily four or five gallons of *Standard Solution No.* 2, which should be made up by the barrel, and kept in a convenient location, for this purpose.

*Disinfection of ingesta.*—It is well established that cholera and typhoid fever, are very frequently, and perhaps usually, transmitted through the medium of infected water or articles of food, and especially milk. Fortunately we have a simple means at hand for disinfecting such infected fluids. This consists in the application of heat. *The boiling temperature maintained for half an hour kills all known disease germs.* So far as the germs of cholera, yellow fever, and diphtheria are concerned, there is good reason to believe that a temperature considerably below the boiling point of water will destroy them. But in order to keep on the safe side it is best not to trust anything short of the boiling point (212° F.) when the object in view is to disinfect food or drink which is open to the suspicion of containing the germs of any infectious disease.

During the prevalence of an epidemic of cholera it is well to boil all water for drinking purposes. After boiling, the water may be filtered, if necessary to remove sediment, and then cooled with *pure* ice if desired.

A sheet of filtering paper, such as druggists use, and a glass or tin funnel, furnishes the best means for filtering water on a small scale for drinking purposes. A fresh sheet of paper is to be used each day.

The above "Preliminary Report" has been prepared at the request of the *Sanitary Council of the Mississippi Valley* as expressed in the following resolution adopted at its recent meeting in the city of New Orleans (March 10-11,

1885).

"Resolved. That the Secretary request from the Chairman of the Committee on Disinfectants, appointed at the last meeting of the American Public Health Association, a plain, practical paper on Disinfection and Disinfectants, for popular use and distribution, to be furnished to the Chairman of the special committee of this Council on General Sanitation."

GEORGE M. STERNBERG, Major and Surgeon U. S. A.
Chairman Committee on Disinfectants.

